# Allogeneic Umbilical Cord Plasma Eyedrops for the Treatment of Recalcitrant Dry Eye Disease Patients

**DOI:** 10.3390/jcm12216750

**Published:** 2023-10-25

**Authors:** Joy Wong, Gayathri Govindasamy, Arun Prasath, William Hwang, Aloysius Ho, Sharon Yeo, Louis Tong

**Affiliations:** 1 Singapore National Eye Centre, 11 Third Hospital Avenue, Singapore 168751, Singapore; joy.wongly@mohh.com.sg (J.W.); gayathri.govindasamy@gmail.com (G.G.); 2 Singapore Cord Blood Bank, 100 Bukit Timah Road, KK Women’s & Children’s Hospital Women’s Tower, Women’s Specialist Clinics, Level 1, Singapore 229899, Singapore; arun.prasath@scbb.com.sg (A.P.); william.hwang.y.k@singhealth.com.sg (W.H.); aloysius.ho.y.l@singhealth.com.sg (A.H.); 3 Department of Haematology, Singapore General Hospital, 31 Third Hospital Avenue, Singapore 168753, Singapore; 4 Singapore Eye Research Institute, 20 College Road Discovery Tower Level 6, The Academia, Singapore 169856, Singapore; sharon.yeo.w.j@singhealth.com.sg; 5 Yong Loo Lin School of Medicine, National University of Singapore, 1E Kent Ridge Road, Singapore 119228, Singapore; 6 Duke-NUS Medical School, 8 College Road, Singapore 169857, Singapore

**Keywords:** dry eye, tear disorder, plasma eyedrops, clinical study, corneal staining, growth factors

## Abstract

Background: Dry eye disease is a significant disease in Singapore. While there have been studies using allogenic cord serum for the treatment of dry eye disease, treatment of dry eyes with allogenic umbilical cord plasma drops has yet to be started in Singapore. Purpose: To evaluate the effectiveness of umbilical cord plasma eyedrops for the treatment of recalcitrant dry eyes in a local Singaporean context. Methods: This is an observational, longitudinal, interventional study for dry eye patients who did not show clear improvement after standard therapy. Patients were recruited from 2020 to 2023 from the dry eye clinic of the Singapore National Eye Center. Umbilical cord plasma was delivered frozen to patients and stored in home freezers. All participants underwent a standardized clinical evaluation for dry eye, and data were collected. Results: There were 40 participants (7 males and 33 females). The duration of follow-up was 5.52 ± 1.57 months. Kerato-epitheliopathy staining score, TBUT (tear breakup time), and SPEED (Standard Patient Evaluation of Eye Dryness Questionnaire) scores significantly improved after treatment. No statistically significant improvement was found in terms of visual acuity, according to Schirmer’s score. Conclusion: Cord plasma eye drops significantly improved kerato-epitheliopathy staining scores in recalcitrant dry eye patients. Allogeneic plasma is a promising form of treatment for recalcitrant dry eye.

## 1. Introduction

Dry eye disease (DED) is a common ocular condition affecting 12.3% of the Singapore population [1,2]. It is a multifactorial disease resulting in ocular discomfort, visual disturbance, and tear film instability. It has been shown to result in functional impairment, especially for activities requiring sustained visual attention (such as reading and driving) [3]. In SNEC (Singapore National Eye Centre), total annual expenditure on dry eye treatment for 2008 and 2009 was USD 1,509,372.20 and USD 1,520,797.80, respectively, contributing significantly to health care expenditure [4].

Ocular surface disorders are characterized by a decrease in the quality and quantity of the tear film and associated changes in the conjunctival and corneal epithelium. The ability to form tears plays an important role in maintaining the health of the ocular surface. The tear film consists of mucous, aqueous, and lipid layers and growth factors, which help to maintain the health and integrity of the ocular surface.

Conventional medical treatments [5] for dry eyes include the application of artificial tears, lid hygiene, topical anti-inflammatory agents, topical corticosteroids, topical cyclosporine A, tacrolimus, macrolides, omega-3 fatty acids, and punctual occlusion. However, there are patients with severe dry eyes [6] who are resistant to the above methods. Thus, new formulations of eye drops are required to treat patients who are unable to find relief with the current options available. Eyedrops derived from blood products (autologous human plasma drops, umbilical cord serum eyedrops, and umbilical cord plasma drops) have a beneficial effect because they have a similar constitution as compared with human tears. They also contain biological factors that promote wound healing and reduce inflammation.

### 1.1. Autologous Human Plasma Eyedrops

There have been studies showing the efficacy of human autologous plasma eyedrops in the treatment of recalcitrant dry eye disease [7]. In the study by Petznick et al., 10 patients with recalcitrant dry eye utilized 100% autologous plasma eyedrops from plasmapheresis. Fifty percent of the patients had improvements in the severity and frequency of dry eye symptoms and reduced corneal staining [8].

However, autologous plasma eyedrops require patients to have patent and adequate-sized veins for cannulation-effective plasmapheresis. The process of obtaining the eyedrops is also time-consuming and costly. Preparing autologous serum eyedrops requires a facility for dilution or aliquoting serum drops into bottles. In many countries, this would require an approved manufacturing facility and trained manpower.

### 1.2. Allogenic Umbilical Cord Blood Eyedrops (Serum/Plasma)

Allogenic umbilical cord blood has higher nerve growth factor (NGF) and substance P concentrations and lower insulin-like growth factor (IGF-1) concentrations compared with plasma blood [9]. Umbilical plasma-supplemented culture medium supports the proliferation and differentiation of epithelial cells in the conjunctiva and limbus. It also contains a higher concentration of growth factors and cytokines than fetal bovine serum and adult serum [9,10]. In addition, it contains bacteriostatic effects due to antibacterial agents such as IgG, lysozymes, and complement [9,10].

Allogenic cord eye drops do not require patients to undergo plasmapheresis and can still be processed within a closed system in an approved pre-existing facility. The drops are prepared by the cord blood bank and delivered directly to patients’ homes, greatly increasing the convenience of the treatment process.

### 1.3. Allogenic Umbilical Cord Serum Eyedrops

Allogenic cord blood serum drops have been applied to treat various ocular surface diseases, including severe dry eye with or without Sjogren’s syndrome, ocular complications in graft-versus-host disease, persistent epithelial defects, neurotrophic keratopathy, recurrent corneal erosions, ocular chemical burns, and surface problems after corneal refractive surgery [9].

### 1.4. Allogenic Umbilical Cord Plasma Eyedrops

While there have been numerous studies investigating the effectiveness of allogenic cord serum eye drops on dry eye patients in Asia [11,12], there has yet to be a study showing the use of closed cord plasma eyedrops for dry eye patients. Plasma, which is usually discarded from the cord blood bank, can now be used to formulate cord plasma eyedrops for the treatment of dry eye disease patients.

The purpose of this article is to provide a prospective longitudinal (interventional cohort) study on the effectiveness and ease of use of cord plasma eye drops in patients with recalcitrant dry eye disease in Singapore. This would be a potential first study using umbilical cord plasma as compared with umbilical cord serum for the treatment of dry eye patients.

## 2. Materials and Methods

### 2.1. Study Design

This is a prospective, longitudinal (cohort), interventional single-arm study.

### 2.2. Study Population

Participants were recruited from the dry eye clinic of the Singapore National Eye Center, a tertiary referral eye center.

All participants provided written informed consent according to hospital regulations concerning treatment with cord plasma eyedrops.

According to the Singapore Biomedical Research Act, studies designed primarily to evaluate treatment efficacy in a clinical service are classified as clinical audits and do not require approval by the institutional review board. Our study complied with the tenets of the Declaration of Helsinki on human research. This report does not reveal identifiable patient data and only uses clinically accepted procedures. No research procedures or assays, such as tear protein analysis, were included, which would require IRB approval and additional consent.

Written informed consent was obtained from the individual(s) for the publication of any potentially identifiable images or data included in this article.

### 2.3. Inclusion Criteria

Participants had to be treated in the same center with the same standard of care (artificial tears, punctal plugs, and immunosuppressives such as topical cyclosporine or corticosteroids). They were considered to have recalcitrant dry eye if no further improvement of corneal staining or symptoms was seen for two consecutive clinic visits in the dry eye clinic. Patients were examined and evaluated by the same single dry eye specialist in the center so as to ensure standardization of assessment for each visit.

### 2.4. Exclusion Criteria

Patients who received surgical intervention (e.g., cataract surgery, corneal transplant) in the midst of treatment were excluded from the study.

### 2.5. Outcome Variables

Primary outcome: the change in corneal epithelial staining at the last visit compared with the first visit.

Secondary outcomes: the standard patient evaluation of eye dryness, SPEED questionnaire, tear breakup times, Schirmer I test, and visual acuity.

### 2.6. Follow-Up Schedule

Each participant was followed up at their first and last visit.

### 2.7. Study Intervention

Plasma preparation protocol:

The Singapore Cord Blood Bank collects donated cord blood (CB) for banking that is potentially used for stem cell transplants. CB is collected in a blood bag containing 35 mL of citrate-phosphate-dextrose-adenine-1 anticoagulant according to validated standard operating procedures by the Singapore Cord Blood Bank (SCBB). The maternal donors signed an informed consent that allows the use of donated cord blood for biomedical research and validation purposes if cord blood units are not eligible for unrelated transplantation. Authorized health care professionals collected cord blood while the placenta was still in utero using validated procedures by SCBB.

**Figure 1:**
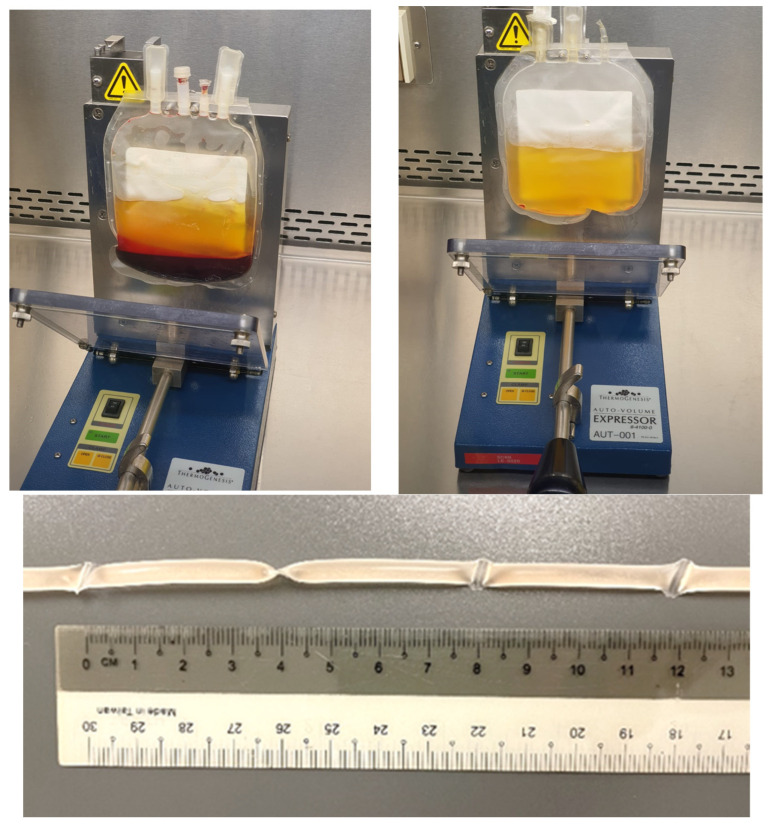
Preparation of umbilical cord plasma.

Cord blood plasma was prepared by the Singapore Cord Blood Bank and distributed to patients. The collected CB units were processed within 48 h of collection for the preparation of platelet-poor plasma. The CB-containing bag was centrifuged at low speed (400× *g*) for 10 min. The platelet-rich plasma (PRP) supernatant was transferred to a secondary bag (Terumo, Japan) using a plasma expressor without disturbing the buffy coat layer and RBC. Please refer to Figure 1 for details of the preparation of umbilical cord plasma.

The bag containing PRP was centrifuged at 2000× *g* for 10 min to pellet the platelets. The supernatant platelet-poor plasma (PPP) was transferred to a new sterile bag without disturbing the platelet pellet. Considering the volume of anticoagulant, an ophthalmologically balanced salt solution (Zeiss) is added to dilute the PPP to achieve 30% plasma in the final product. The diluted plasma was filled in sterile intravenous tubes (Terumo) and sealed as single-use segments of approximately 4 cm each.

The PPP-containing segments were stored in a −18 °C freezer overnight before being transferred to a −80 °C freezer for long-term storage or delivered frozen to patients for use and storage in home freezers.

The safety of the product was evaluated by serology for infectious disease markers in maternal blood (for HIV, HBV, HCV, and syphilis) and nucleic acid testing (for HIV, HBV, and HCV). For sterility testing, samples from PPP and RBC-containing bags were used to determine the presence of aerobic and anaerobic bacteria and fungi (BD Bactec). Only samples that tested negative for microbiology and infectious diseases were distributed to patients.

Patients used plasma drops after thawing each day in addition to any prescribed eyedrops. The patients were instructed to administer plasma treatment at frequencies of one to three times a day in both eyes.

The mean frequency of use among patients was 2.23 times a day. Eight participants used cord plasma once a day, 14 participants used it twice a day, six used it three times a day, and four used it more than four times a day. Eight participants did not indicate the frequency of drops used. Depending on the number of vials of plasma used per day (one to three per day), each lot of plasma would last for two to four months.

### 2.8. Co-Existing Treatment

The same treatment before the commencement of the allogeneic plasma drops was continued during the study period. Refer to Supplementary Tables S1–S3 for the types of co-existing treatments that patients were on during cord plasma treatment.

### 2.9. Details of Study Procedure

SPEED Questionnaire

All patients underwent symptom evaluation with the previously validated standard patient evaluation of eye dryness (SPEED) questionnaire, which scored the frequency and severity of the following symptoms: dryness, grittiness, scratchiness, irritation, burning, watering of the eyes, soreness, and eye fatigue. The questionnaire then scored patients from 0 to 28, which assessed the frequency and severity of the eight symptoms mentioned above. Scores from all sub-questions were added, and the greater the total score (0–28), the more frequent or severe the dry eye [13,14,15].

Tear breakup time (TBUT)

A drop of normal saline was instilled on a fluorescein strip (Fluorets) and then shaken off so that no visible drop remained. A fluorescein dye was applied to the inferior fornix of the patient’s eye, and the patient was instructed to blink and close their eyes. The patient’s tear film was then observed under cobalt blue illumination on the slit lamp. The tear break-up time (TBUT) was measured as the time that elapsed between the last blink and the appearance of the first dry spot in the tear film [16].

Fluorescein-dyed corneal staining

For corneal fluorescein staining, we used a grading method for superficial punctate keratopathy by Miyata et al. After staining the eye with a minimal amount of sodium fluorescein dye (saline-moistened fluoret), the total area of staining was graded from A0 to A3, and the density was graded from D0 to D3. The grading was represented as the combination of the area and density grades [17].

Schirmer’s test

Schirmer test was performed with the standard 5 mm wide Test Strips (Haag-Streit Clement Clarke Intl. (Essex, UK)) with a notch for folding without prior anesthesia. The strips were positioned over the inferior temporal half of the lower lid margin in both eyes, and participants’ eyes were then closed. The extent of the wetting was recorded after 5 min [18].

Visual acuity

Two versions of the modified LogMAR chart were used routinely in the Singapore National Eye Center. The visual acuity was recorded and converted to Snellen notation.

### 2.10. Statistical Methods

Continuous data were analyzed by paired T tests.

Since we did not have a previous study on cord plasma eyedrops, we did not have effect sizes to calculate sample sizes. However, we ensured that the study exceeded the number of patients published in a similar study on cord serum (*n* = 31) [12].

Since the right and left eyes were not statistically independent, we used only data for one eye per patient: the eye with more improvement in clinical signs over time. However, only one eye from each patient was used in the statistical analysis (*n* = 40). The report presented the improvement in the right and left eye data separately.

Statistical improvement was at the level of alpha = 0.05.

## 3. Results

The study recruited 40 participants (7 males and 33 females) with a mean age of 68.01 ± 12.00 years. The mean duration of follow-up was 5.52 ± 1.57 months. Supplementary Tables S1 and S2 show the co-existing treatments for participants during the study period.

The underlying etiology of dry eye was as follows: Ten had Sjogren’s syndrome, seven had glaucoma, five had graft versus host disease, three had rheumatoid arthritis, and two had LASIK.

**Figure 2:**
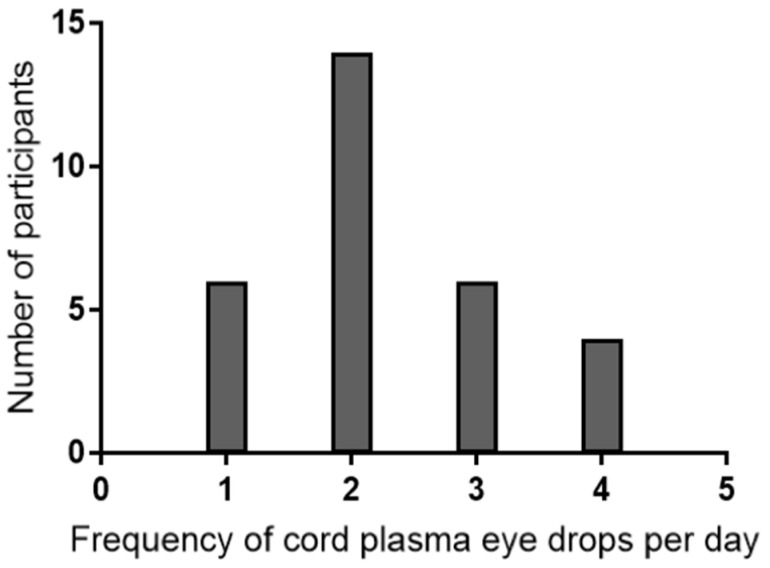
Frequency of cord plasma eyedrops per day.

Figure 2 shows the frequency of cord plasma drops used per participant.

Table 1 shows the percentage change in parameters. Parameters that improved in more than 50% of patients included corneal punctate staining, tear breakup time, SPEED score, and Schirmer’s test. In terms of punctate staining, 81% improved and 8% worsened. In terms of visual acuity (VA), 28% improved, 46% remained the same, and 26% worsened. The TBUT of 51% of participants improved, whereas in 22%, this worsened, with a mean change of +0.62. The SPEED score improved in 58% and worsened in 31%, with a mean change of −4.14. The Schirmer’s test score improved by 50% and worsened by 23%, with a mean change of −0.45.

Table 2 shows the change in parameters pre- and post-treatment. Kerato-epitheliopathy staining score significantly decreased after treatment compared with baseline (3.92 ± 2.75 vs. 1.97 ± 1.56, *p* < 0.001). TBUT also significantly increased after treatment compared with baseline (1.70 ± 0.87 vs. 2.29 ± 1.21, *p* = 0.02). The SPEED score showed significant improvement after treatment compared with baseline (10.54 ± 7.56 vs. 6.4 ± 5.42, *p* = 0.01). No statistically significant improvement was found in terms of VA or Schirmer’s score. (15.25 ± 13.95 vs. 13.29 ± 6.25, *p* = 0.58, and 4.52 ± 6.11 vs. 4.07 ± 5.02, *p* = 0.75, respectively).

No eye infections or adverse effects from the eye drops were noted.

### 3.1. Case Reports

Written informed consent was obtained from the individuals for the publication of any potentially identifiable images or data included in this article.

#### 3.1.1. Case 1

**Figure 3:**
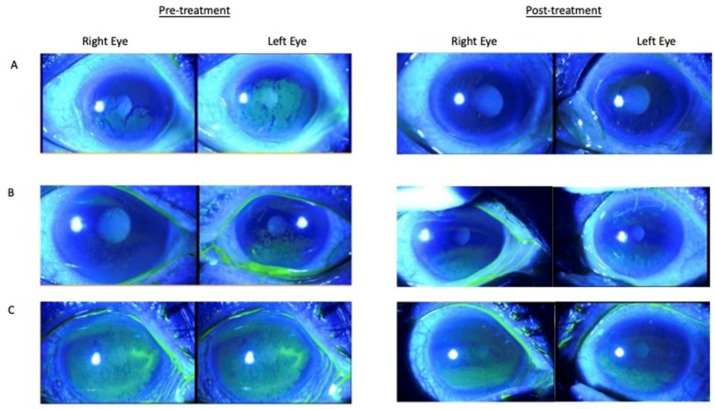
Case studies (**A**): Case 1. Corneal staining photos for pre-treatment and post-treatment with 6 months of cord plasma. Her corneal epitheliopathy staining score was 2 and 9 in her right and left eye, respectively, pre-treatment; post-treatment, her score was 0 in both eyes. Her TBUT improved from RE 2s LE 2s to RE 5s LE 3d post-treatment. Her SPEED score improved from 17 to 0 post-treatment. (**B**): Case 2. Corneal staining photos for pre-treatment and post-treatment with 4 months of cord plasma. Her corneal epitheliopathy staining score was 1 and 3 in her right and left eye, respectively, pre-treatment; post-treatment, her score was 1 and 2. Her SPEED score improved from 26 to 6 post-treatment. (**C**): Case 3. Corneal staining photos pre-treatment and post-treatment with 4 months of cord plasma. His corneal epitheliopathy staining score was 9 and 9 in his right and left eye, respectively, pre-treatment; post-treatment, his score was 3 and 3. His SPEED score improved from 8 to 0 post-treatment.

This was a 50-year-old patient (Figure 3A) with a past medical history of primary Sjogren’s syndrome (1990), dry eyes, and previous punctal plugging (2019). Her other medical history included end-stage renal failure with secondary tubulonephritis, anemia, bronchiectasis, fibrocystic breast cyst, cervical spondylosis, hypertension, and hyperlipidemia. Her current dry eye treatment included topical immunosuppressive drops and neurostimulation with Trutears, artelac lipids TDS, Vidisc, loteprednol BD, olopatadine BD, and tears naturale-free eyedrops Q3H. She complained of persistent bilateral blurring of vision and dry eyes.

#### 3.1.2. Case 2

This was a 51-year-old patient (Figure 3B) with a past medical history of hypothyroidism on thyroxine, previous LASIK, and blepharitis in both eyes. She was treated with bilateral punctal plugs, vidisc, ciclosporin 0.1% nocte, copious g refresh plus, thera tears eye lid cleanser, lotemax eyedrops with a tapering dose, levofloxacin, and duratears ointment.

#### 3.1.3. Case 3

This was a 60-year-old patient (Figure 3C) with a past medical history of dry eyes and primary open-angle glaucoma. His current treatment included bilateral punctal plugs, cyclosporine, and lotemax eyedrops. He was also treated with Ganfort and Azopt eyedrops for glaucoma.

## 4. Discussion

Our study shows that umbilical cord plasma eyedrops are a promising therapy for patients with recalcitrant dry eye disease. Prior to starting cord plasma, our cohort of patients had achieved no further improvement with their pre-existing dry eye treatment. After a course of cord plasma, our patients showed promising results, with the corneal epitheliopathy staining score significantly improving from 3.92 ± 2.75 to 1.97 ± 1.56, *p* < 0.001. Other clinical parameters, like tear breakup time, also improved from 1.70 ± 0.87 to 2.29 ± 1.21, *p* = 0.02. Subjectively, patients’ symptom score improved from 10.54 ± 7.56 to 6.4 ± 5.42, *p* = 0.01.

While there have been multiple studies showing the effectiveness of cord serum for dry eye patients, this would be a potential first study using umbilical cord plasma as compared with umbilical cord serum for the treatment of dry eye patients.

The main differences between plasma and serum are as follows:

Serum requires aliquoting into bottles and diluting it before dispensing. Furthermore, serum is the fluid that remains after blood has clotted and would require removal of the clot by transferring to a tube for centrifuging or similar techniques, which means additional handling of fluid. As this is not performed in a closed system, there is greater concern about infection and the potential for the addition of antibiotics. Since the bottles used are 10 mL, there are also issues with contamination and protein degradation in the absence of freezing.

Plasma can be used at very high concentrations as it is diluted only by the anticoagulant used. This ensures optimal efficacy due to the concentration of growth factors and anti-inflammatory factors.

The aliquoting and handling of the serum would be considered manufacturing in Singapore and need to be done in a facility approved for this purpose. On the other hand, the running of cord plasma in a closed bag and IV tube and crimping the IV tube fall within the approved operational procedures in the stem cell bank. So for regulatory reasons, we can currently only use the plasma approach.

Our treatment results with cord plasma eye drops show similar outcomes with patients treated with cord serum. In a study by Yoon et al., which assessed 55 eyes of 31 patients with severe dry eye syndrome treated with cord serum eyedrops in Korea, there was significant improvement in several outcomes. Symptoms score, tear breakup time, keratoepitheliopathy score, and impression cytologic findings improved significantly after application of umbilical cord serum drops [12]. Similarly, our study also reported significant improvements in the keratoepitheliopathy score, tear breakup time, and symptoms scoring.

It is noteworthy that we have obtained similar conclusions despite differences in methodology. Yoon’s paper graded symptom scores on a numerical scale of 0 to 4, with 0 representing no symptom, 1 representing mild symptoms that did not constitute discomfort, and 4 representing very severe symptoms that caused discomfort and interfered with normal activities. Our paper used the Standard Patient Evaluation of Eye Dryness questionnaire to evaluate the symptoms of our patients.

Our studies also had differing methods in terms of the preparation of umbilical cord serum eyedrops. In Yoon’s paper [12], umbilical cord blood serum was diluted to a concentration of 20% with an unpreserved normal saline solution. The diluted serum was then aliquoted into sterile 5ml bottles with ultraviolet light protection. The bottles were kept in the refrigerator and valid for use for up to 3 months. For our study, we used 30% umbilical cord plasma diluted with citrate anticoagulant and balanced salt solution (BSS), which was then filled in sterile IV tubes and crimped into single-use segments. Cord plasma was prepared in crimps/ individual vials that were kept in the patients’ home freezers. When required, they were warmed to room temperature for one-time use. Our novel method of eye drop preparation ensures sterility with single-use individual segments that reduce the risk of contamination and infection.

In another case series by Susiyanti et al. (2022), which assessed the efficacy of cord serum eyedrops for dry eyes in ocular Stevens–Johnson syndrome, improvements in symptoms scores, Schirmer scores, and keratoepitheliopathy scores were noted after treatment [19]. This is also consistent with our cohort, which showed improvement in dry eye symptoms, keratoepitheliopathy score, and Schirmer’s scoring after treatment with cord plasma eyedrops, though the etiology of dry eyes was different in our study population.

Umbilical cord-derived fluid contains growth factors such as epithelial growth factor, transforming growth factor-β, and insulin-like growth factor, providing an optimal ocular surface environment [12]. These factors contribute to epithelial cell proliferation and migration, encouraging corneal epithelial healing. EGF can promote cell proliferation due to its anti-apoptotic activity [20]. Insulin-like growth factor facilitates corneal regeneration and plays a role in stem cell growth [21]. n a randomized controlled clinical trial by Vajpayee et al., umbilical cord serum was compared with autologous serum drops to promote the healing of persistent corneal epithelial defects (PED). Patients treated with cord serum showed greater improvement as compared with those treated with autologous serum [22]. In another prospective case control study by Yoon et al. [11], which compared the therapeutic effect between autologous serum and umbilical cord serum eye drops in the treatment of severe dry eyes, umbilical cord serum eye drops were shown to be superior to autologous serum eyedrops in terms of decreasing symptoms and keratoepitheliopathy. These studies are consistent with our results, which showed significant improvement in corneal epithelial staining and symptoms after treatment with cord plasma.

In addition to a high concentration of growth factors, cord blood plasma also contains proliferative and immunomodulatory factors such as TGF-β, G-CSF, GM-CSF, monocyte chemoattractant protein (MCP)-1, IL-6, and IL-8. Cytokines such as TGF-β have an important function in stem cell proliferation and differentiation. G-CSF and GM-CSF have also been shown to mobilize stem cells [23,24]. These factors might contribute to the mobilization of corneal limbal stem cells that aid in the regeneration of the corneal surface.

This is the first study to assess the use of umbilical cord plasma drops for recalcitrant dry eye. A novel method of umbilical cord plasma eyedrop preparation was used, which involved preparing cord plasma in single-use segments to ensure sterility. Our results have shown that cord plasma has promising treatment outcomes for dry eye patients and have shown consistent results with prior studies done with cord serum.

### 4.1. Limitations

As this was a prospective observational study, we did not standardize the concurrent dry eye therapy or the frequency of cord plasma used. There might also be variable amounts of exosomes or platelet products between different cord plasma batches.

The patients were recruited in a consecutive manner using cord plasma drops. Thus, when the study stopped, they would have been on treatment for different durations of time. Longer-term follow-up would be more informative, and this longitudinal audit will continue for these and future patients.

There was also no randomization since there is only one intervention arm. In our setting, where dry eye patients are recalcitrant to standard treatment, the maximal therapy for each patient is different, and they have different underlying etiologies. Hence, we were unable to select a single type of therapy as a control. No other additional type of intervention is available in our context once standard therapy has been maximized. However, the results of this current study can guide future research and pave the way for more robust controlled trials, as we would know the treatment effect size for sample size calculation in comparative trials.

### 4.2. Further Potential Areas of Research

Umbilical cord plasma can also be combined with a PROSE (prosthetic replacement of the ocular surface ecosystem) lens for constant exposure to growth factors. Used in conjunction with PROSE, a rigid prosthetic device with a fluid reservoir, cord plasma can be used to provide an ideal ocular environment for optimal ocular surface healing and regeneration [25].

Other potential areas of research include the viability of long-term storage and the potential benefits of adding platelet lysates.

There is also a potential for using umbilical cord plasma in regenerative therapy [21]. Umbilical cord plasma can be considered for other clinical uses, such as in optic nerve regeneration and cell replacement therapy for retinal pathologies. A preliminary study that assessed cord serum drops administered to glaucoma patients showed improvement in visual field test parameters, possibly due to the high growth factor content in the serum, which might confer a neuroprotective effect on the optic nerve [26].

Cord plasma is potentially low-cost, as the plasma, which is usually discarded by the blood bank, is repurposed as eyedrops. There is also no need to perform additional donor tests for infection as they have already been tested because of cord stem cell banking regulations. Cord plasma can be stored in the refrigerator and does not require special storage facilities.

## 5. Conclusions

In conclusion, cord plasma eyedrops are a promising and effective therapy for recalcitrant dry eyes in Singapore. Patients showed significant improvement in kerato-epitheliopathy staining score, tear breakup time, and dry eye symptom score after treatment.

## Figures and Tables

**Table 1:** Change in clinical parameters of the patients.

%	VA	TBUT	Schirmer’s	Corneal Epitheliopathy Score	SPEED
Improved	28	51	50	81	58
Same	46	27	23	11	11
Worsened	26	22	27	8	31

**Table 2:** Change in parameters pre- and post-treatment.

Parameter	First Visit (Before Treatment)	Post Treatment	Change (Last Visit-First Visit)	*p*-Value
SPEED	10.54 ± 7.56	6.4 ± 5.42	−4.14	0.01
TBUT (s)	1.70 ± 0.87	2.29 ± 1.21	+0.59	0.02
VA (logMAR letters)	15.25 ± 13.95	13.29 ± 6.25	−1.96	0.58
Kerato-epitheliopathy staining score	3.92 ± 2.75	1.97 ± 1.56	−1.95	0.001
Schirmers (mm)	4.52 ± 6.11	4.07 ± 5.02	−0.45	0.75

## Data Availability

The data presented in this study are available on request from the corresponding author. The data are not publicly available due to patient confidentiality.

## Supplementary Materials

The following supporting information can be downloaded at: https://www.mdpi.com/article/10.3390/jcm12216750/s1, Table S1: co-existing dry eye treatment; Table S2: use of artificial tears and non prescription eyedrops; Table S3: Concomitant dry eye treatment per patient.
