# Supplementary material for: Allogeneic Umbilical Cord Plasma Eyedrops for the Treatment of Recalcitrant Dry Eye Disease Patients
Source: J Clin Med. 2023 Oct 25;12(21):6750. doi: 10.3390/jcm12216750 (PMC10648694; doi:10.3390/jcm12216750)
Supplement: Supplementary file 1 [file jcm-12-06750-s001.zip › Supplementary Table S1.pdf]

Supplementary Table S1: Co-existing dry eye treatment

	Co-existing treatment	No of participants
1	Lotemax	10
2	Dexamethasone	1
3	Ikervis	7
4	Ciclosporin 0.5%	5
5	Ciclosporin 1%	1
6	Restasis	2
7	Diquafosol	15
8	Punctal Plug	1
