# Supplementary material for: Allogeneic Umbilical Cord Plasma Eyedrops for the Treatment of Recalcitrant Dry Eye Disease Patients
Source: J Clin Med. 2023 Oct 25;12(21):6750. doi: 10.3390/jcm12216750 (PMC10648694; doi:10.3390/jcm12216750)
Supplement: Supplementary file 1 [file jcm-12-06750-s001.zip › Supplementary Table S2.pdf]

Supplementary Table S2: Use of artificial tears and non-prescription eyedrops

	Co-existing eyedrops	No of participants
1	Tears naturale free	17
2	Vidisic gel	16
3	Duratears	10
4	Hialid or hialid plus	1
5	Optive	3
6	Refresh or Refresh plus	8
7	Hypromellose or EyeMo	2
8	Systane ultra	4
9	Artelac or soothe o	2
10	Cationorm	1
11	Olopatadine	3
