# Supplementary material for: Allogeneic Umbilical Cord Plasma Eyedrops for the Treatment of Recalcitrant Dry Eye Disease Patients
Source: J Clin Med. 2023 Oct 25;12(21):6750. doi: 10.3390/jcm12216750 (PMC10648694; doi:10.3390/jcm12216750)
Supplement: Supplementary file 1 [file jcm-12-06750-s001.zip › Supplementary Table S3.pdf]

Supplementary Table S3: Concomitant dry eye treatment per patient

case	Gender	Age	Treatment used
1	F	50	artelac lipids<br>vidisc<br>loteprednol<br>olopatadine<br>tears naturale preservative free (TNPF)
2	F	75	ciclosporin 0.1%<br>Lotemax (loteprednol etabonate 0.5%)<br>Duratears<br>TNPF
3	F	62	acetylcysteine 5% QDS<br>blephagel<br>vidisic<br>loteprednol etabonate 0.5% BD<br>TNPF Q3H
4	F	51	lotemax tapering dose<br>vidisc gel<br>ciclosporin 0.1% BE<br>refresh plus<br>Duratears<br>Punctal plugs BE
5	F	73	vidisic<br>ciclosporin 0.1%<br>thera tears capsule<br>Optive fusion

7	M	60	punctal plugs<br>cyclosporine 0.5%<br>lotemax<br>duratears<br>optive fusion
6	M	88	ciclosporin 0.5%<br>diquafosol<br>TNPF<br>vidisic
8	M	68	Systane ultra eye drop<br>Vidisic gel
9	F	85	diquafosol<br>blephagel<br>vidisic<br>ikervis
10	F	67	lotemax<br>diquafosol
11	F	61	lotemax
12	F	71	diquafosol<br>refresh plus<br>blephagel
13	M	90	diquafosol<br>dexamethasone (minims) ciclosporin 0.5%
14	F	79	diquasfosol

15	F	84	TNPF<br>Duratears ointment<br>ciclosporin 0.5%<br>vidisc gel
16	F	62	TNPF<br>vidisic gel<br>diquafosol<br>fucithalmic<br>vidisic gel
17	M	73	thera tears capsule<br>ciclosporin 0.05%<br>refresh 1%<br>duratears
18	M	55	refresh<br>lotemax<br>vidisic<br>diquafosol
20	M	78	ciclosporin 0.1%<br>cravit 0.5%<br>dexamethasone minims<br>refresh
21	F	70	TNPF<br>lotemax<br>diquafosol

22	F	51	TNPF<br>solcoseryl 20% eye gel diquafosol<br>ciclosporin 1%<br>vidisic<br>blephagel
23	M	67	optive fusion<br>restasis<br>theratears capsules<br>lotemax LE<br>dexamethasone 0.1% minims RE<br>bandage Contact Lens<br>punctal plug
24	F	55	diquafosol<br>lotemax<br>vidisic<br>TNPF<br>refresh
25	M	60	ikervis<br>TNPF
26	F	60	lubricants, blephagel, couldnt tolerate<br>diquas (stopped)
27	M	75	TNPF<br>lotemax<br>ciclosporin<br>punctal plugs
28	F	65	TNPF<br>lotemax

29	F	81	TNPF<br>diquafosol<br>cyclosporine<br>lotemax<br>punctal plugs
30	F	62	diquafosol<br>TNPF<br>duratears
31	F	71	TNPF
32	F	68	diquafosol<br>patanol<br>vidisc gel
33	F	78	diquafosol<br>TNPF<br>vidisic gel
34	F	60	diquafosol<br>eyemo
35	F	66	ikervis<br>TNPF
36	F	51	Hialid mini<br>Soothe XP<br>ikervis<br>artelac lipids<br>lotemax
37	F	53	Diquafosol<br>TNPF<br>Duratears ointment<br>Vidisic gel
38	F	74	TNPF

39	F	72	systane ultra<br>patanol<br>ikervis<br>FML
40	M	34	Cationorm<br>manuka honey eyedrops
41	F	69	systane ultra eye drops<br>duratears<br>ciclosporin
42	M	86	refresh<br>diquafosol<br>vidisic gel<br>ikervis
43	F	87	
44	F	70	optive fusion eye drops<br>diquafosol
45	F	80	systane ultra eye drops<br>diquafosol
46	F	83	TNPF<br>duratears
47	F	73	diquafosol<br>ikervis
48	F	56	TNPF
49	F	56	diquafosol<br>refresh
50	F	69	Acetylcysteine<br>TNPF<br>Duratears ointment

51	F	58	Eyemo
52	F	58	TNPF<br>lotemax
53	M	90	TNPF
54	F	72	diquafosol<br>refresh<br>ikervis
55	F	72	
56	F	67	lotemax<br>diquafosol
57	F	79	
58	M	83	refresh plus<br>vidisic<br>refresh plus
59	F	61	diquafisol<br>TNPF
60	F	86	refresh
61	M	64	diquafosol<br>omega 3 capsules
62	F	59	diquafosol<br>vidisic gel
63	F	77	TNPF
